# Guided intraoperative scintigraphic tumor targeting of metastatic cervical lymph nodes in patients with differentiated thyroid cancer: a single-center report

**DOI:** 10.20945/2359-3997000000068

**Published:** 2018-10-01

**Authors:** Ethem Turgay Cerit, Mehmet Muhittin Yalçin, Çiğdem Ӧzkan, Müjde Aktürk, Alev Eroğlu Altinova, Ümit Ӧzgür Akdemir, Murat Akin, Emre Arslan, Ayhan Karakoç, Ali Riza Çimen, Nuri Çakir

**Affiliations:** 1 Gazi University Gazi University Faculty of Medicine Department of Endocrinology and Metabolism Ankara Turkey Department of Endocrinology and Metabolism, Faculty of Medicine, Gazi University, Ankara, Turkey; 2 Gazi University Gazi University Faculty of Medicine Department of Nuclear Medicine Ankara Turkey Department of Nuclear Medicine, Faculty of Medicine, Gazi University, Ankara, Turkey; 3 Gazi University Gazi University Faculty of Medicine Department of General Surgery Ankara Turkey Department of General Surgery, Faculty of Medicine, Gazi University, Ankara, Turkey

**Keywords:** Differentiated thyroid cancer, guided intraoperative scintigraphic tumor targeting, radio-guided occult lesion localization, recurrent thyroid carcinoma

## Abstract

**Objective:**

Our aim was to present our experiences related to performing neck surgery using the guided intraoperative scintigraphic tumor targeting (GOSTT) procedure for patients who had locally recurrent or persistent differentiated thyroid cancer (DTC) and who had undergone previous thyroid surgery.

**Subjects and methods:**

We retrospectively evaluated 11 patients who had locally recurrent or persistent DTC, who had undergone previous surgery, and for whom reoperation was planned for metastatic cervical lymph nodes (LNs). We performed the neck surgery using the GOSTT procedure on all patients and at a single academic institution.

**Results:**

The 11 patients had a total of 26 LNs, as marked with a radiotracer, and those LNs’ mean size was 14.7 ± 8.2 mm (range: 5-34 mm). Histopathological examinations revealed DTC metastasis in all 26 of the preoperatively marked LNs. Of the 11 patients, only one needed a reoperation in the neck; she had another successful surgery (also using the GOSTT procedure). In the evaluation of the patients’ final status, all were disease-free in their necks. There also were no GOSTT-associated postoperative complications.

**Conclusion:**

The GOSTT procedure is a useful, successful, inexpensive, and comfortable procedure for marking and mapping metastatic LNs, especially in DTC patients who have undergone previous surgery.

## INTRODUCTION

Globally, thyroid cancer is the most common endocrine neoplasia (1). Differentiated thyroid cancer (DTC) is the most frequent thyroid cancer subtype (2). Although DTC has an excellent outcome, with the use of standard pathological techniques, cervical lymph node (LN) metastases have been detected in 20 to 50% of patients in most series. LN metastases may be present even when the primary tumor is small and/or has an intrathyroidal location (3,4). During follow-ups, using neck ultrasonography (US) and monitoring thyroglobulin (Tg) levels can lead to greater identification of nonpalpable LN metastases in the central and lateral neck, particularly in patients who have been newly diagnosed or who have undergone previous thyroid surgery (5).

Various surgical approaches exist for lateral cervical LN dissection (6). Selective lateral LN dissection is the preferred surgical approach, as it does not lead to higher morbidity and minimizes local recurrence rates (6-9). In the “berry picking” procedure, which has been recommended for the local recurrence of LN metastases in areas that have been previously dissected, only suspicious and/or enlarged lymph nodes are removed (6). Biopsy-proven metastatic LNs in a compartment that previously underwent surgery should be considered for excision, but reoperations are associated with increased morbidity compared with primary tumor surgery (10,11). Reoperative surgery in the neck for recurrent DTC is a burdensome and demanding procedure for surgeons because of the presence of scarred and fibrotic tissues in that area (11).

Radio-guided surgery (RGS) is a popular modality in surgical practice. RGS allows a surgeon to identify lesions or tissues that have been preoperatively marked with radioactive substances (12). RGS is performed using either systemic or local injection of radioisotopes. Radio-guided occult lesion localization (ROLL), a type of GOSTT, was originally described for occult lesions in breast cancer (13). Recently, GOSTT has also been performed on patients who have locally recurrent or persistent DTC and who are undergoing a repeat neck surgery (5,12,14-24). ROLL is based on the intralesional injection of a radioactive substance that (because of its large size) does not migrate from the site of the interstitial injection. In the ROLL technique, the radiation that other tissues absorb is negligible. Therefore, this technique is presumably more advantageous than systemic radionuclide administration (15,16). In ROLL, a surgeon confirms complete removal of a lesion by repeatedly counting the surgical bed with the gamma probe. All of the published results indicate that GOSTT-associated radiation exposure to both health care professionals and patients is minimal. The radiation exposure doses for patients, GOSTT administrators, surgeons, nurses, and pathologists are all within the safety limits recommended by the International Commission on Radiological Protection (14). Routine monitoring of radiation exposure after the GOSTT procedure is not recommended for any nonnuclear medical professionals (14).

Our aim in this study was to present our experiences and results related to performing the GOSTT procedure on patients who had locally recurrent or persistent DTC and who had undergone previous thyroid surgery.

## SUBJECTS AND METHODS

We retrospectively evaluated 11 patients who underwent total thyroidectomy with or without central and/ or lateral LN dissection; these patients all had locally recurrent or persistent DTC and all had previously undergone thyroid surgery. Reoperation was planned for all 11 patients’ metastatic cervical LNs to address 1 or more suspicious LNs that had been detected via neck I US. We confirmed the diagnosis of LN metastasis due I to DTC via US-guided fine needle aspiration biopsy I (FNAB) and monitoring of the FNAB-Tg washout level (10). We then performed the repeat neck surgery using the GOSTT procedure for all patients.

### GOSTT procedure

We percutaneously injected the radioactive particles directly into the lesion 1 to 2 h prior to surgery. Specifically, we used a 22-gauge needle and a syringe to inject 0.2 mCi of technetium-99m human albumin macroaggregate aliquot (Makro-Albumon, Medi- Radiopharma, Budapest, Hungary) in a volume of 0.1 ml suspension directly into each suspicious lymph node using 7.5 MHz linear transducers (GE Logiq 5 Pro system equipped with an 8-12 MHz linear probe, GE Medical Systems, Milwaukee, WI) under continuous US monitoring. We withdrew the needle under slight aspiration in order to minimize the release of residual radioactivity along the needle track. We marked the skin directly overlying each suspicious LN with a surgical skin marker under US guidance. A nuclear medicine specialist had previously prepared the radioactive material. The nuclear medicine department enforced radiation safety protocols, and we performed the procedure in the nuclear medicine department. The marked cutaneous projections of the lesions on the neck skin provided a useful guide for deciding upon the appropriate incision site at the beginning of surgery. During surgery, we used a handheld gamma-detection probe (Crystal CXSSGV2 model, Crystal Photonics, Berlin, Germany) that was covered by a sterile probe cover to locate the suspicious lesions and decide upon the most appropriate excision of the radio-labeled lesions. We excised the premarked LNs along with any adjacent suspicious LNs or soft tissues in order to accomplish the selective LN dissection. We used the berry-picking procedure for patients who had undergone previous compartment- oriented dissections in the current suspicious region. After excision of all premarked and suspicious LNs, we monitored the suspicious area for residual radioactivity in each patient.

We performed the statistical analyses (in terms of mean, range, ratio, and percent) using SPSS version 16.0 (Chicago, IL) software.

We obtained informed consent from all patients before performing the FNAB and GOSTT procedures. The local ethics board approved this retrospective study.

## RESULTS

Eleven patients who had recurrent or persistent DTC and who had undergone previous thyroid surgery were included to this study. The mean age of the patients was 41.6 ± 18.3 years (range: 20-71 years). The female-to-male ratio was 5:6. The patients’ tumor, node, and metastasis classifications, cancer stages, and histopathological characteristics are shown in Table 1. Each of the patients had undergone at least 1 surgery (range: 1-3) prior to the GOSTT procedure. The types of prior operations are also shown in Table 1. Eight patients had undergone radioactive iodine ablation before the GOSTT procedure.

**Table 1:** Patient and tumour characteristics

Patient no.	Sex	Age at Diagnosis (yr)	TNM classification	Stage	Histology of carcinoma	Operation(s) before RGS	No. of neck operation before RGS	Total I-131 dose before RGS
1	M	31	T1bN1aM0	Stage 1	Classical type PTC	TT, CLND	1	150
2	F	36	T1bN1bM0	Stage 1	Classical type PTC	TT, CLND, LLLND	2	118
3	F	23	T2N1aM0	Stage 1	Classical type PTC	TT, CLND	1	125
4	M	35	T1bN1bM1	Stage 2	Classical type PTC	TT, CLND, RLLND, LLLND	3	150
5	F	55	T3N1bM0	Stage 4a	Follicular variant PTC	TT	1	50
6	F	20	T3N1bM0	Stage 1	Diffuse sclerosing variant PTC	TT, CLND, RLLND	2	100
7	M	71	T3N1bM0	Stage 4a	Classical type PTC	TT	1	0
8	M	60	T1aN1bM0	Stage 4a	Follicular variant PTC	TT, CLND	1	0
9	F	27	T3N1bM1	Stage 2	Classical variant PTC	TT, LLLND	2	100
10	M	68	T2N1bM1	Stage 4c	Widely invasive FTC	TT, LLLND, RLLND	2	150
11	M	32	T1bN1aM0	Stage 1	Classical variant PTC	TT, CLND	1	0

M: male; F: female; No: number; PTC: papillary thyroid cancer; FTC: follicular thyroid cancer; LN: lymph node; TNM: tumour, node, metastasis; RGS: radioguided surgery; TT: total thyroidectomy; CLND: cantral LN dissection; RLND: right lateral LN dissection; LLND: left lateral LN dissection; RAI: radioactive iodine treatment.

A total of 26 LNs were marked for the GOSTT procedure across the 11 patients. The mean size of the marked LNs was 14.7 ± 8.2 mm (range: 5-34 mm). The sites of the lesions are shown in Table 2. No complications were encountered during the procedure. All the patients underwent successful excisions, even for lesions of less than 1 cm. We performed berry-picking in the 5 patients who had undergone previous compartment-oriented dissections in the current suspicious region (Patients 1, 2, 3, 10, and 11). Histopathological examinations revealed DTC metastasis in all of the 26 preoperatively marked LNs. There were no postoperative complications (Table 2).

**Table 2:** Characteristics of guided intraoperative scintigraphic tumour targeting procedure and follow up of patients after radioguided surgery

Patient no.	Site of LN in GOSST procedure (mm)	Size of LN in GOSST Procedure (mm)	Type of operation with GOSST procedure	No. of marked LN with GOSST procedure	No. of metastatic LN found	No. of total excised LN	Pre RGS (TSH,Tg, Anti-T)	Post RGS (early period) (TSH, Tg, Anti-T)	Compl	Distant metastasis	Tx after RGS	Follow up time after RGS (mo)	Final status in neck	Total follow up time (mo)	Final disease status
1	Left level VI	11	BP In left level VI	2	4	5	TSH: 1.5 Tg: 0.8 Anti -T: N	TSH: 0.2 Tg: 0.2 Anti -T: N	None		150 mCi I 131	48	Neck US negative TSH:0.05 Tg:< 0.2 Anti-T: N	72	LDF
2	Left level VA	14	BP In left level VA	1	1	3	TSH: 156 Tg: 65 Anti -T: N	TSH: 0.3 Tg: 0.2 Anti-T: N	None		150 mCi 1-131	36	Neck US negative TSH: 0.01 Tg: 0.2 Anti-T: N	54	LDF
3	Left level VI	8	BP In left level VI	2	4	4	TSH: 11 Tg: 3.9 Anti -T: N	TSH: 147 Tg: 1.6 Anti-T: N	None		150 mCi 1-131	28	Neck US negative TSH: 0.05 Tg: 0.2 Anti-T: N	82	LDF
4	Right level IV	34	RLLND	4	10	10	TSH: 100 Tg: 266 Anti -T: N	TSH: 110 Tg: 168 Anti-T: N	None	Lung, mediastinal LN	400 mCi 1-131, mediastinal LND	36	Neck US negative TSH: 0.01 Tg: 0.2 Anti -T: N	48	LDF
5	Left level VI and left level III	9	CLND, LLLND	2	2	18	TSH: 0.8 Tg: 0.4 Anti -T: P	TSH: 116 Tg: < 0.2 Anti-T: N	None		150 mCi 1-131	24	Neck US negative TSH: 0.01 Tg:< 0.2 Anti-T: N	40	LDF
6	Right level VI and right level III	25	CLND, RLLND	3	6	12	TSH: 324 Tg: 8.4 Anti -T: N	TSH: 74 Tg: 1.4 Anti -T: N	None		150 mCi 1-131	50	Neck US negative TSH: 0.01 Tg: 0.2 Anti-T: N	69	LDF
7	Right level VI and right level IV	9	CLND, RLLND	2	2	22	TSH: 74 Tg: 33 Anti-T: N	TSH: 121 Tg: 5.3 Anti -T: N	None		150 mCi 1-131	15	Neck US negative TSH:0.01 Tg: 0.2 Anti-T: N	18	LDF
8	Right level lll-IV-V	11	RLLND	3	4	20	TSH: 0.5 Tg: 3.3 Anti -T: P	TSH:0.01 Tg: 0.2 Anti -T: N	None		150 mCi 1-131	24	Neck US negative TSH: 0.02 Tg:< 0.2 Anti-T: N	27	LDF
9	Left level VI and left level lll-IV	9	CLND, LLLND	3	3	11	TSH: 227 Tg: 230 Anti -T: P	TSH: 149 Tg: 56 Anti -T: P	None	Lung	400 mCi 1-131, LLLND with ROLL	18	Neck US negative TSH: 0.01 Tg: 11 Anti -T: N	30	LWD
10	Right level VI and right level IV	20	BP In right level VI and level IV	2	2	4	TSH: 116 Tg: 208 Anti -T: N	TSH: 100 Tg: 500 Anti -T: N	None	Lung, mediastinal LN	600 mCi 1-131 Mediastinal LND+TCi?	42	Neck US negative TSH: 0.05 Tg: 145 Anti-T: N	85	LWD
11	Left level VI and right level VI	L-12mm R-5mm	BP In left and right level VI	2	2	2	TSH:0.14 Tg:<0.2 Anti -T: P	TSH: 104 Tg: 6.7 Anti -T: P	None		150 mCi 1-131	10	Neck US negative TSH:0.02 Tg< 0.2 Anti-T: N	12	LDF

LN: lymph node; BP: berry picking; GOSST: guided intraoperative scintigraphic tumour targeting; CLND: cantral LN dissection; RLND: right lateral; LN dissection; LLND: left lateral LN dissection; LDF: living disease free; LWD: living with disease; TCI: tyrosine kinase inhibitor; Tg: thyroglobulin (ng/mL); TSH: thyroid stimulating hormone (μIU/mL); Tx: treatment; RGS: radioguided surgery; RAI: radioactive iodine treatment; Compl: complication; Anti-T: anti-thyroglobulin antibody; P: positive (high); N: negative (in normal range).

All of the patients received postoperative radioactive iodine ablation treatment. The mean follow-up time after GOSTT was 30.0 ± 13.3 months (range: 10-50 months). Of the 11 patients, only 1 had recurrent disease in the neck, and she underwent another successful RGS to remove these metastatic lymph nodes (Patient 9). During the follow-up, 3 patients developed distant metastasis (in the lungs and the mediastinal LNs); two underwent mediastinal LN dissection as a result (Patients 4 and 10). At the final patient-status evaluation, all of the patients were disease-free in their necks, but 2 patients (Patients 9 and 10) were still living with disease because of distant metastasis to the lungs (Table 2).

## DISCUSSION

Although thyroid cancer mostly follows an indolent course, the patients in our study had late-stage disease or required recurrent operations. All patients in this study had preoperative skin markings done via the GOSTT procedure and underwent successful excisions, even for lesions less than 1 cm. Serum Tg levels decreased in 8 of 11 patients during the early postoperative period. At the final patient-status evaluation, all of the patients were disease-free in their necks.

Compared to primary thyroid surgery, reoperation for locally recurrent or persistent DTC carries increased risks for damage to both the recurrent laryngeal nerve and the parathyroid glands. For reoperative procedures, the incidence of permanent vocal cord paralysis is 1-12% and the incidence of hypoparathyroidism is 1-4% (11,15,25). Even though recurrent neck surgery seems more difficult than primary surgery, the patients in our study experienced no GOSTT-associated postoperative complications. The operative time was also shorter than expected (operative time data is not available). The GOSTT procedure could be comfortable for surgeons during the incision and excision processes. Using a gamma probe also helps surgeons to confirm that all suspicious LNs have been excised.

Tükenmez and cols., using the ROLL technique, reported the first successful resection of nonpalpable LN metastases in 2 DTC patients (17). Three prospective studies were published in 2010, all of which indicated that the ROLL technique was safe and effective for the removal of nonpalpable DTC metastases (18-21). Terzio**ğ**lu and cols. reported the excision of 30 lesions from 21 patients undergoing reoperative thyroid and parathyroid surgery, all without any nerve injury or transient hypoparathyroidism. Tg levels decreased to less than 2 ng/ml in 86% of the patients with preoperatively elevated Tg levels (16). Gulcelik and cols. reported on 20 patients with metastatic DTC who underwent the ROLL technique with a 100% success rate and with no postoperative complications other than a single seroma (22). Borsò and cols., in a study of 32 patients, found no cervical DTC recurrences (all tests were radioiodine-negative) after performing surgery with the ROLL technique (26). In our cases, all of the 26 suspicious LNs revealed DTC metastasis on histopathologic examination. Of the 11 patients, only one needed a reoperation in the neck; she underwent another successful surgery (also using the GOSTT procedure).

The injection of a radioactive substance into a suspicious LN is technically similar to LN FNAB. Clinicians who can perform LN FNAB should be able to easily inject a radioactive substance into suspicious LNs.

In conclusion, preoperative mapping with US-guided intralesional radiotracer injection and intraoperative gamma-probe use, as compared to conventional methods, allows for faster intraoperative detection of metastatic lesions, could facilitate the surgical approach, and could improve surgical outcomes for patients who have locally recurrent or persistent DTC and who have previously undergone neck surgery. Thus, GOSTT is a convenient, comfortable, and inexpensive procedure for selected patients with locally recurrent or persistent DTC.
